# LLM evaluation for thyroid nodule assessment: comparing ACR-TIRADS, C-TIRADS, and clinician-AI trust gap

**DOI:** 10.3389/fendo.2025.1667809

**Published:** 2025-09-29

**Authors:** Xi Dai, Yu Xi, Yong Hu, Qingyan Ding, Yu Zhang, Hui Liu, Piaofei Chen, Xi Wang, Wenjun Wang, Chaoxue Zhang

**Affiliations:** ^1^ Department of Ultrasound, The First Affiliated Hospital of Anhui Medical University, Hefei, Anhui, China; ^2^ Department of Ultrasound, Huangshan City People’s Hospital, Huangshan, Anhui, China; ^3^ Department of Endocrinology, Huangshan City People’s Hospital, Huangshan, Anhui, China; ^4^ Department of Thyroid, Breast and Hernia Surgery, Huangshan City People’s Hospital, Huangshan, Anhui, China

**Keywords:** large language models (LLMs), thyroid nodules, risk stratification, ACR-TIRADS, C-TIRADS, clinical decision-making

## Abstract

**Objective:**

To evaluate the diagnostic performance and clinical utility of advanced large language models (LLMs) -GPT-4o, GPT-o3-mini, and DeepSeek-R1- in stratifying thyroid nodule malignancy risk and generating guideline-aligned management recommendations based on structured narrative ultrasound descriptions.

**Methods:**

This diagnostic modeling study evaluated three LLMs—GPT-4o, GPT-o3-mini, and DeepSeek-R1—using standardized narrative ultrasound descriptors. These descriptors were annotated by consensus among three senior board-certified sonologists and processed independently in a stateless manner to ensure unbiased outputs. LLM outputs were assessed under both ACR-TIRADS and C-TIRADS frameworks. Two experienced clinicians (a thyroid surgeon and an endocrinologist) independently rated the outputs across five clinical dimensions using 5-point Likert scales. Primary outcomes included the area under the receiver operating characteristic curve (AUC) for malignancy prediction, and clinician ratings of guideline adherence, patient safety, operational feasibility, clinical applicability, and overall performance.

**Results:**

GPT-4o achieved the highest predictive AUC (0.898) under C-TIRADS, approaching expert-level accuracy. DeepSeek-R1, particularly with C-TIRADS, received the highest clinician ratings (mean Likert: surgeon 4.65, endocrinologist 4.63), reflecting greater trust in its practical recommendations. Clinicians consistently favored the C-TIRADS framework across all models. GPT-4o and GPT-o3-mini received lower ratings in trustworthiness and recommendation quality, especially from the endocrinologist.

**Conclusion:**

While GPT-4o demonstrated superior diagnostic accuracy, clinicians most trusted DeepSeek-R1 combined with the C-TIRADS framework for generating practical, guideline-consistent recommendations. The findings highlight the critical need for alignment between AI-generated outputs and clinician expectations, and the importance of incorporating region-specific clinical guidelines (like C-TIRADS) for the effective real-world implementation of LLMs in thyroid nodule management decision support.

## 1 Introduction

Thyroid nodules are a common clinical finding with most being benign but a small proportion harboring malignant potential. The increasing prevalence of thyroid nodules, largely due to the widespread high-resolution ultrasonography (1, 2), underscores the need for accurate risk stratification. This guides clinical management by minimizing unnecessary invasive procedures and optimizing patient outcomes (3).

Recent advancements in the diagnosis and management of thyroid nodules have been driven by both refined risk stratification systems and the integration of artificial intelligence (AI). We selected ACR-TIRADS (4) and C-TIRADS (5) for their reproducibility and alignment with global (ACR-TIRADS) and regional Chinese clinical guidelines (C-TIRADS). We prioritized these over Eu-TIRADS, which has fewer risk categories that may limit nuanced evaluations in our cohort (6). Large language models (LLMs) excel in thyroid nodule assessment by processing unstructured narratives. They simulate expert reasoning and generate guideline-aligned recommendations. This differs from traditional AI methods like S-Detect, which rely on image-based feature extraction without interpretive depth (3, 6, 7). A review by Grani et al. (3) outlines current diagnostic and therapeutic strategies for thyroid nodules. It underscores AI’s growing role in enhancing risk assessment accuracy, particularly via ultrasound-based systems like ACR-TIRADS and C-TIRADS. For instance, Multimodal GPT systems show promise in improving diagnostic performance and reducing unnecessary biopsies and surgeries. In this study, we focused on the 2017 American College of Radiology Thyroid Imaging Reporting and Data System (ACR-TIRADS) as a global comparator and the 2020 Chinese Thyroid Imaging Reporting and Data System (C-TIRADS) as our routine clinical standard. This reflects international familiarity and local decision-making thresholds. These systems are widely adopted: ACR-TIRADS in the US and C-TIRADS in China. This ensures our findings’ relevance to the investigated clinical settings. Other guidelines, like Eu-TIRADS and K-TIRADS, use different biopsy triggers and lexicons. These could affect LLM recommendations. Future work will evaluate these guidelines to assess generalizability. LLMs, such as GPT-4o, excel in analyzing both structured and unstructured clinical narratives. They offer advantages in complex scenarios requiring detailed textual analysis. In contrast, traditional AI systems like S-Detect focus primarily on structured image data. This may limit their adaptability in complex clinical scenarios (8). Furthermore, Yang et al. (6) corroborate the need for integrated approaches. Multimodal systems, including LLMs, demonstrate superior diagnostic accuracy in thyroid nodule evaluation compared to traditional image-based AI systems. C-TIRADS shows favorable performance among TIRADS systems and S-Detect. This capability positions LLMs as valuable tools for complementing image-based decision-making in complex scenarios (7).

Building on these advancements, LLMs—including OpenAI’s GPT series and models like DeepSeek-R1—demonstrate promise in healthcare applications (9–11). These models process structured imaging data and unstructured narratives. They simulate expert reasoning and generate evidence-based management recommendations. However, the clinical validity and reliability of LLMs in TIRADS frameworks remain underexplored and unvalidated in real-world settings. This validation gap prompted our investigation into LLMs’ potential for thyroid nodule management.

To address this gap, we evaluated LLMs’ role in thyroid nodule management using two approaches. First, we compared the diagnostic performance of three LLMs (GPT-4o, GPT-o3-mini, and DeepSeek-R1) against expert sonologists under ACR-TIRADS and C-TIRADS. Second, we assessed model outputs across five key clinical dimensions: guideline adherence, patient safety, operational feasibility, clinical applicability, and overall performance. These evaluations provide insights into LLMs’ strengths and limitations in endocrine workflows. They inform strategies for safe clinical integration.

To our knowledge, this is the first study to evaluate LLMs under dual TIRADS systems with structured trust assessments from experts. Our findings provide foundational insights into integrating LLMs into thyroid nodule management pathways.

## 2 Materials and methods

### 2.1 Study design and ethical approval

This retrospective, single-center study was conducted at our hospital and approved by the institutional ethics committee. Due to the study’s retrospective nature, informed consent was waived. The sample size of 63 nodules was appropriate for this preliminary study. It is comparable to other initial AI evaluations in thyroid disease management, such as a study using 33 patient queries that detected significant differences (P < 0.01) in performance metrics. This provides sufficient power (approximately 70-80%) to detect meaningful differences in AUC (0.05-0.1) based on similar comparisons.

### 2.2 Patient cohort

We initially reviewed 150 adult patients with thyroid nodules. After applying exclusion criteria, we included 93 patients who underwent thyroidectomy from January 2020 to October 2024. Exclusion criteria were:

- Incomplete clinical records
- Prior thyroid surgery history
- Inadequate or low-quality ultrasound images
- Absence of preoperative ultrasound performed at the study institution
- Inconsistent nodule characteristics interpretation among three senior sonologists

**Figure 1:**
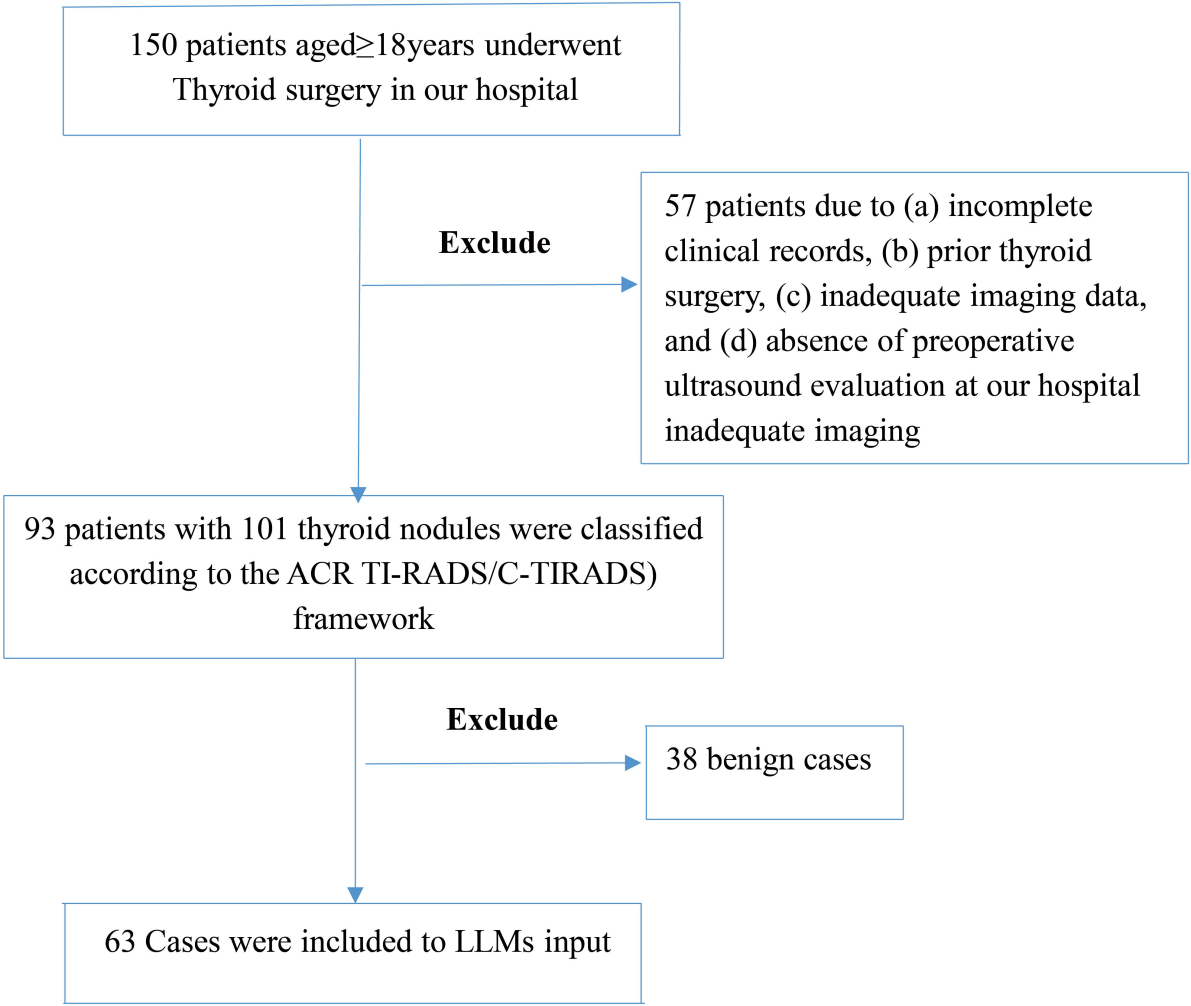
Patient selection flowchart. This figure outlines the inclusion and exclusion steps applied to the thyroid nodule ultrasound case pool. After applying clinical and imaging eligibility criteria, 63 cases were retained for analysis. The process excludes 57 patients followed by exclusion of 38 benign cases, resulting in a subset enriched for diagnostic ambiguity to stress-test models.

To account for multifocal disease and heterogeneous histopathology, we analyzed 101 distinct nodules from these 93 patients. The cohort included 30 males and 63 females, with both solitary and multifocal nodules. The patient selection process is illustrated in **Figure 1**.

### 2.3 Data collection

Two board-certified sonologists, each with over 10 years of thyroid imaging experience, independently extracted clinical and ultrasound data. We implemented a two-phase review protocol to ensure ultrasound annotation consistency. Before data extraction, all sonologists underwent a calibration session. This standardized interpretation criteria based on the 2017 ACR-TIRADS and 2020 C-TIRADS guidelines (5, 9, 11).All three sonologists had comparable training and experience in thyroid ultrasound interpretation.

Key assessed ultrasound features included:

- Composition
- Echogenicity
- Margins
- Shape
- Calcifications
- Vascularity
- Central and lateral lymph node status

In the first phase, the two sonologists independently assessed all nodules. Inter-observer agreement was measured using Cohen’s kappa coefficient. Nodules with substantial agreement (κ ≥ 0.60) across all features proceeded to the second phase. In the second phase, a third board-certified sonologist independently reassessed eligible nodules. This sonologist was blinded to prior evaluations and all clinical/pathological data. We included only nodules with unanimous agreement among all three sonologists on predefined features in the final dataset. This strict consensus process maximized annotation reliability and minimized inter-observer variability. It ensured high-fidelity input for LLM analysis.

Collected data included:

- Demographics and medical history
- Thyroid function tests (TSH, T3, T4, autoantibodies)
- Detailed ultrasound features
- Postoperative histopathology

### 2.4 LLM prompt construction and output generation

We crafted structured prompts using standardized clinical and imaging data. These were refined through iterative pilot testing and sonologist consensus to ensure clarity, consistency, and relevance. We tested three prompt variations to optimize phrasing for model performance. These aligned with the 2017 ACR-TIRADS and 2020 C-TIRADS guidelines. The initial prompt was overly rigid. It used numbered steps, fixed scoring, and predefined outputs. This constrained dynamic reasoning and flexibility, risking repetitive, biased responses that overlooked nuances. We refined prompts iteratively over three cycles using a pilot set of 10 nodule descriptions. We tested variations like basic inputs (e.g., “Are you familiar with the 2017 ACR-TIRADS guidelines?/Are you familiar with the 2020 C-TIRADS guidelines? Analyze this ultrasound description: [details]. Classify using ACR-TIRADS by scoring features and provide category/risk.”) versus structured formats. These included TI-RADS criteria, role prompts (e.g., “act as an expert”), and output templates to enhance consistency and reduce ambiguity. We applied a unified prompt template consistently across all three LLMs (GPT-4o, GPT-o3-mini, and DeepSeek-R1). It used identical formats and parameters for fair comparison. We used uniform optimized prompts across all models for fair comparison. Example: “Please act as an expert in thyroid nodules. Analyze the patient’s clinical data and ultrasound features. Classify according to the 2017 ACR-TIRADS and 2020 C-TIRADS. Assign malignancy risk and provide management recommendations.” Each prompt represented a single thyroid nodule. It incorporated demographic data, laboratory values, and ultrasound features. Prompts were written in natural language to simulate real-world clinical narratives. Example: “Act as an expert in thyroid nodules. Based on the ACR-TIRADS (2017), classify this nodule: [description]. Assign a malignancy risk level and provide management recommendations.” We processed prompts independently in a stateless framework. This prevented prior context from influencing responses and enhanced reproducibility and fairness.

To focus on challenging cases, we excluded 38 unequivocally benign nodules. These were confirmed by histopathology and uniformly classified as low-risk across all models. Excluded cases showed no ambiguous features. We removed them to focus analysis on borderline or complex presentations. We intentionally enriched the analytic subset for ambiguous cases. This increased malignancy prevalence (73% vs. 45.5% in the total cohort). This strategy rigorously tested model performance in complex cases.

Interpret findings from this focused sample with caution, especially in routine screening settings. We plan prospective sampling at routine prevalence to validate findings and improve generalizability.

The final dataset included 63 nodules:

- 17 multifocal cases (15 malignant)
- 6 with lateral lymph node metastasis (LLNM)
•21 with central lymph node metastasis (CLNM)

Although this yielded a higher-than-average malignancy rate, the enrichment enabled rigorous testing of model performance in complex cases.

### 2.5 Expert evaluation of LLM outputs

Two independent experts -a thyroid surgeon and an endocrinologist, both experienced in thyroid disease management- evaluated each model’s recommendations. They used a 5-point Likert scale across the following five domains:

- Guideline adherence (ACR-TIRADS, C-TIRADS)
- Patient safety (conservativeness and appropriateness)
- Operational feasibility (ease of clinical implementation)
- Clinical utility (usefulness in real-world decision-making)
- Overall performance

To minimize bias, we anonymized and blinded model outputs to the source model and true clinical outcomes. We assessed inter-rater reliability using weighted Cohen’s kappa (quadratic weights) on merged Likert scores (1–2: Low, 3: Medium, 4–5: High). This was done for each dimension, separately for ACR-TIRADS and C-TIRADS, and per model. It evaluated consistency between surgeon and endocrinologist ratings **(Supplementary Table S3**).

### 2.6 Selection criteria for large language models

We selected LLMs based on:

- Strong performance in general reasoning and medical benchmarks
- Release within the past two years
- Accessibility via public APIs or open platforms
- Architectural diversity (e.g., proprietary vs. independently trained models)

Specifically:

- GPT-4o and GPT-o3-mini: Represent OpenAI’s GPT serie**s** (12, 13).
- DeepSeek-R1: Developed independently by DeepSeek (14).

### 2.7 Statistical analysis

Descriptive statistics summarized patient demographics and characteristics.

We assessed diagnostic performance via:

- Cohen’s kappa for agreement between LLM classifications and sonologist consensus
- Sensitivity, specificity, PPV, NPV, and AUC (histopathology as reference standard)
- ROC curve analysis for performance visualization.
- We used 95% confidence intervals for proportions via the Wilson method (z=1.96). AUC comparisons used the DeLong procedure. Cohen’s κ CIs used bias-corrected bootstrap (1,000 resamples). Point estimates are reported to three decimals places. Results are in **Table 1**.
- We adjusted PPV and NPV for different prevalences (5%, 10%, 15%) using Bayesian methods. Adjustment results are in **Supplementary Table S1**.
- We evaluated model performance separately for subgroups based on nodule size, malignancy status, and lymph node metastasis. Subgroup analyses calculated AUC, sensitivity, specificity, PPV, NPV, and 95% CIs. AUC comparisons used the DeLong test. Subgroup results are in **Supplementary Table S2**.

**Table 1:** Diagnostic performance of AI models and expert readers based on ACR-TIRADS and C-TIRADS guidelines.

Model	Guideline	Sensitivity (95% CI)	Specificity (95% CI)	PPV (95% CI)	NPV (95% CI)	AUC (95% CI)
Expert	ACR-TIRADS	1.000 [0.923, 1.000]	0.800 [0.676, 0.884]	0.807 [0.687, 0.889]	1.000 [0.920, 1.000]	0.900 [0.848, 0.952]
Expert	C-TIRADS	1.000 [0.923, 1.000]	0.745 [0.617, 0.842]	0.767 [0.646, 0.856]	1.000 [0.914, 1.000]	0.874 [0.815, 0.929]
GPT-4o	ACR-TIRADS	0.957 [0.855, 0.988]	0.800 [0.676, 0.884]	0.800 [0.676, 0.884]	0.957 [0.855, 0.988]	0.879 [0.815, 0.934]
GPT-4o	C-TIRADS	0.870 [0.743, 0.939]	0.927 [0.827, 0.971]	0.909 [0.788, 0.964]	0.895 [0.789, 0.951]	0.898 [0.835, 0.957]
GPT-o3-mini	ACR-TIRADS	0.891 [0.770, 0.953]	0.855 [0.738, 0.924]	0.837 [0.710, 0.915]	0.904 [0.794, 0.958]	0.873 [0.803, 0.935]
GPT-o3-mini	C-TIRADS	0.957 [0.855, 0.988]	0.691 [0.560, 0.797]	0.721 [0.598, 0.818]	0.950 [0.835, 0.986]	0.824 [0.758, 0.886]
DeepSeek-R1	ACR-TIRADS	0.783 [0.644, 0.877]	0.836 [0.717, 0.911]	0.800 [0.662, 0.891]	0.821 [0.702, 0.900]	0.809 [0.731, 0.882]
DeepSeek-R1	C-TIRADS	0.957 [0.855, 0.988]	0.709 [0.579, 0.812]	0.733 [0.610, 0.829]	0.951 [0.839, 0.987]	0.834 [0.764, 0.896]

Values are shown as point estimate [95% CI]. 95% CIs for sensitivity, specificity, PPV, and NPV were computed using the Wilson method (z=1.96), and 95% CIs for AUC were derived with the DeLong approach. Cohort composition: n_malignant = 46 and n_benign = 55. PPV/NPV depend on disease prevalence.

We included only nodules with full consensus from three sonologists to ensure ground truth reliability.

We calculated inter-observer agreement between the two initial readers using Cohen’s kappa. Nodules with κ ≥ 0.60 proceeded to third-round review. Final inclusion required full agreement across all features.

We tested expert Likert ratings for normality using the Shapiro–Wilk test. Depending on distribution, we applied one-way ANOVA or Kruskal–Wallis tests. We considered a two-tailed p-value < 0.05 statistically significant.

All analyses used Python (v3.9.18).

## 3 Results

Our preliminary analyses showed moderate concordance between LLMs and sonologist classifications. We focused subsequent analyses on inter-model comparisons and expert evaluations of clinical applicability.

### 3.1 Patient characteristics

From January 2020 to October 2024, we initially collected 150 adult thyroid nodule cases from a tertiary medical center. After rigorous quality assessment and review by three sonologists, we included 101 nodules from 93 patients in the final analysis (inclusion rate: ~ 67.3%). This stringent selection ensured diagnostic relevance and data integrity.

The median patients was 52.0 years (range: 42.0–58.0). Of the 93 patients, 30 (32.3%) were male and 63 (67.7%) were female. Postoperative histopathology confirmed 46 (45.5%) malignant and 55 (54.5%) benign nodules. Baseline clinical characteristics are summarized in **Table 2**, which compares demographics and clinical features of the full cohort (101 nodules from 93 patients) and the annotated subset (63 nodules) to assess representativeness.

**Table 2:** Baseline characteristics of the study population and comparative statistics between the total cohort and expert-annotated subset.

Characteristic	Total (n = 101)	Subset (n = 63)	P-value
Number of patients	93	63	–
Number of nodules	101	63	–
Gender, n (%)			0.754
Female	63 (67.7%)	45 (71.4%)	
Male	30 (32.3%)	18 (28.6%)	
Age (y)			0.666
Mean ± SD	49.9 ± 12.5	48.7 ± 12.5	
Median (IQR)	52.0 (42.0–58.0)	52.0 (37.5–58.0)	
Range	19–78	25–75	–
Maximum nodule diameter (mm)			0.129
Mean ± SD	19.5 ± 17.1	17.0 ± 16.5	
Median (IQR)	13.0 (7.9–23.0)	9.9 (6.7-18.0)	
Range	3.0–73.0	4–72	–
Pathological results, n (%)			<0.001**
Benign	55 (54.5%)	17(27.0%)	
Malignant	46 (45.5%)	46 (73.0%)	

Data are presented as mean ± standard deviation (SD), median (interquartile range [IQR]), range, or number (percentage), as appropriate. Continuous variables were compared using the Mann–Whitney U test; categorical variables were analyzed using the chi-square (χ²) test. A two-tailed P-value < 0.05 was considered statistically significant.

### 3.2 Data quality and model input integrity

To ensure high-quality, reliable LLM input, ultrasound data underwent rigorous annotation. Two board-certified sonologists, each with over 10 years of experience, independently annotated all ultrasound features relevant to TIRADS classification. Inter-observer agreement was strong (Cohen’s κ = 0.81). A third board-certified sonologist, with equivalent qualifications and over 10 years of experience, independently and blindly re-evaluated all cases to ensure consistency and minimize annotation bias. We retained only nodules with unanimous agreement on key features (echogenicity, composition, shape, margin, and calcifications), enhancing interpretive consistency and clinical validity.

To focus on diagnostically meaningful cases, we excluded 38 unequivocally benign nodules with consistent low-risk categorization across all models. The remaining 63 nodules had complex, borderline, or ambiguous features, enabling robust assessment of model reasoning and decision-making under uncertainty.

For LLM input, we converted structured annotations into concise, standardized clinical narratives.

These included patient demographics, relevant clinical history, and key ultrasound descriptors (location, size, echogenicity, margin, shape, calcifications, vascularity, and lymph node status). For example:

“A 41-year-old female presented with a left-sided neck mass detected one week ago. Thyroid function test shows TSH at 5.5 uIU/ml, with no other abnormalities.

Ultrasound findings: No evidence of diffuse thyroid disease. The nodule, located in the mid-portion of the left lobe, measures approximately 18 mm × 15.4 mm × 16 mm. It is solid, hypoechoic, exhibits capsular invasion, ill-defined margins, angular borders, and a taller-than-wide shape. Microcalcifications are present. Grade 2 internal vascularity is observed. Multiple abnormal lymph nodes with irregular shapes and rich vascular flow are identified in levels 2, 3, 4, and 6 on the left side.

These prompts emulated routine physician documentation, enabling LLMs to process cases using natural clinical language rather than abstracted templates. Additional examples are in Appendix 1.

We presented each case to the LLMs independently in a stateless manner. This ensured outputs were generated solely from each prompt, unaffected by prior cases. This design enabled unbiased evaluation of model diagnostic reasoning.

### 3.3 Bayesian-adjusted predictive values

To account for the enriched malignancy prevalence (73% in subset vs. typical 5–15%), we adjusted PPV and NPV using Bayesian methods (**Supplementary Table S1**). Across models and TIRADS, adjusted NPV was high (>0.96; e.g., GPT-4o C-TIRADS: 0.979–0.994), indicating effective malignancy exclusion and biopsy reduction in routine settings. Adjusted PPV was moderate (0.22–0.65; e.g., DeepSeek-R1 C-TIRADS: 0.24–0.514), suggesting potential false positives in low-prevalence settings, consistent with reported thyroid nodule malignancy rates of 7–15% (7)).

### 3.4 Diagnostic concordance between LLMs and expert grading

Under the ACR-TIRADS framework, both GPT-4o and GPT-o3-mini showed substantial agreement with expert annotations by board-certified sonologists (Cohen’s κ = 0.614 [0.490, 0.738]). DeepSeek-R1 showed moderate concordance (κ = 0.480 [0.358, 0.597]).

Under C-TIRADS, GPT-o3-mini had the highest agreement (κ = 0.368 [0.249, 0.474]), followed by DeepSeek-R1 (κ = 0.223 [0.129, 0.322]) and GPT-4o (κ = 0.212 [0.112, 0.318]), all indicating fair agreement levels.

**Figure 2:**
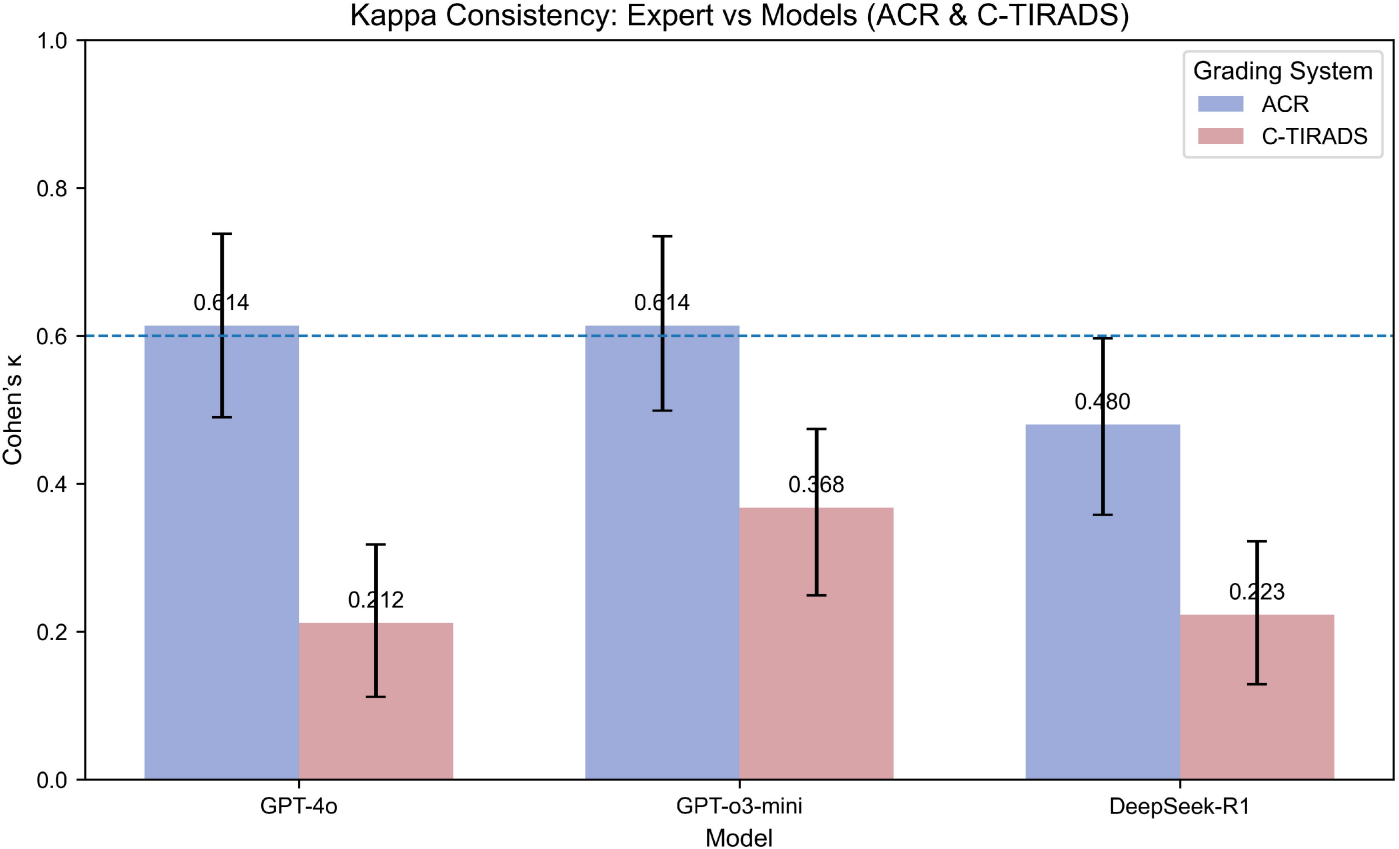
Kappa consistency between expert and models (ACR & C-TIRADS grading systems). This bar plot compares the Kappa coefficients for model consistency with expert grading using both the ACR and C-TIRADS systems. Higher Kappa values indicate stronger agreement between the model and the expert. The models evaluated include GPT-4o, GPT-o3-mini, and DeepSeek-R1. The Kappa coefficient is presented for each model under both grading systems to show the degree of alignment between AI predictions and expert evaluations. “Expert” refers to the board-certified sonologist. Kappa values (with 95% CI via percentile bootstrap) indicate fair-to-substantial agreement, with GPT-4o highest under ACR-TIRADS (κ=0.614), reflecting framework-specific alignment strengths.

Kappa statistics are in **Figure 2**.

### 3.5 Diagnostic performance for malignancy prediction

ROC analysis showed the board-certified sonologist, using ACR-TIRADS, achieved the highest malignancy diagnostic accuracy (AUC = 0.900; 95% CI: 0.848–0.952).

Among LLMs, GPT-4o with C-TIRADS had the highest AUC (0.898; 95% CI: 0.835–0.957), slightly outperforming its ACR-TIRADS variant (AUC = 0.879; 95% CI: 0.815–0.943). GPT-o3-mini with ACR-TIRADS had an AUC of 0.873 (95% CI: 0.803–0.935), while DeepSeek-R1 with ACR-TIRADS had the lowest (AUC = 0.809; 95% CI: 0.731–0.882).

**Figure 3:**
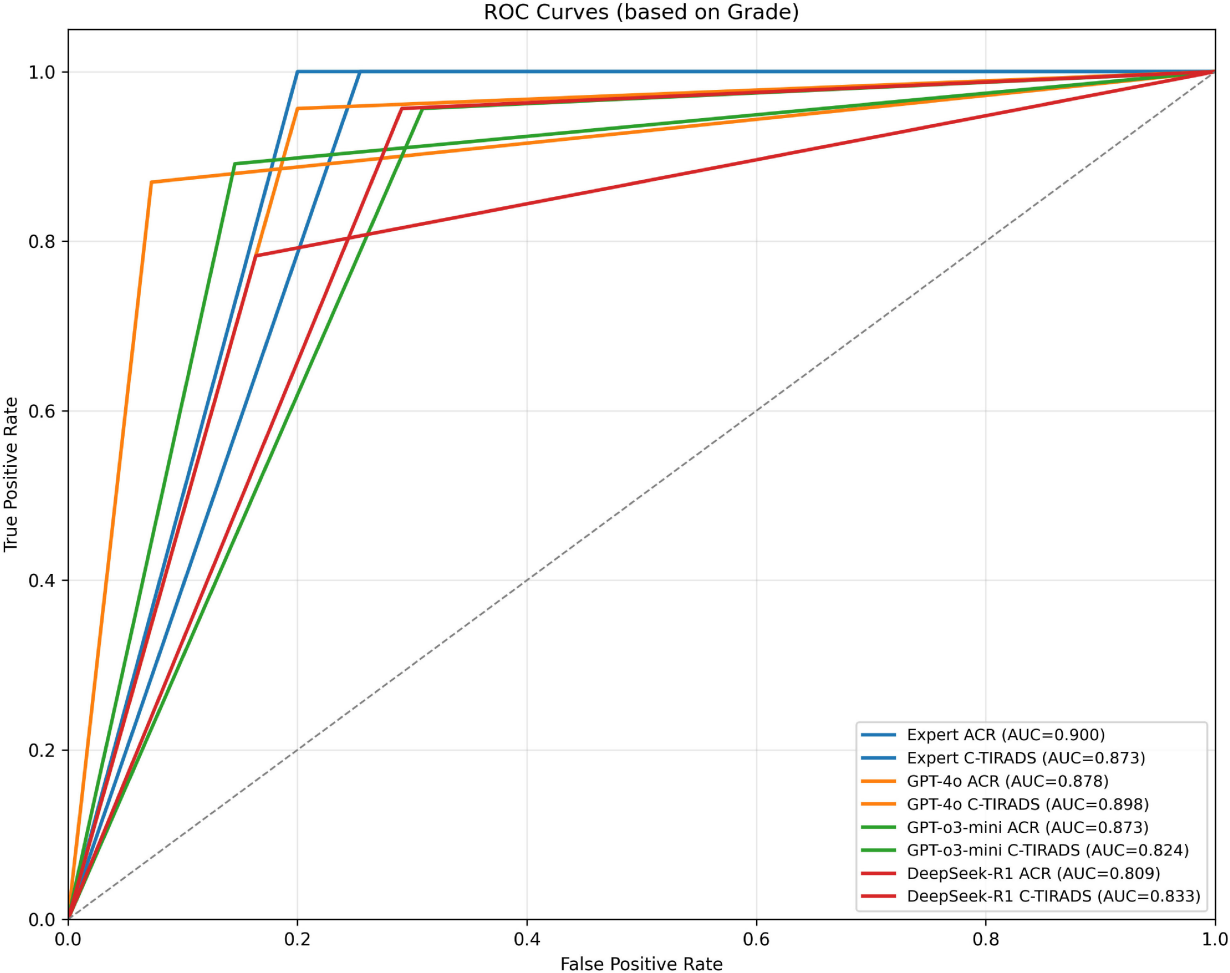
ROC curves for expert and models based on grading systems (ACR & C-TIRADS). Receiver Operating Characteristic (ROC) curves illustrating the diagnostic performance of expert assessments and AI models under the ACR and C-TIRADS grading systems. Each curve represents the True Positive Rate (sensitivity) versus False Positive Rate (1-specificity) at different thresholds. The Area Under the Curve (AUC) for each model is shown in parentheses to quantify overall diagnostic accuracy. Models evaluated include GPT-4o, GPT-o3-mini, and DeepSeek-R1. “Expert” refers to the board-certified sonologist. AUC near 0.9 (e.g., GPT-4o C-TIRADS: 0.898) shows expert-level malignancy prediction, with better performance under C-TIRADS, indicating its suitability for AI integration.

We performed a *post-hoc* DeLong test to compare AUC values between GPT-4o and DeepSeek-R1 under ACR-TIRADS and C-TIRADS. The test showed that, although GPT-4o had higher AUC values than DeepSeek-R1, the differences were not significant (p= 0.893 for ACR-TIRADS, p= 0.875 for C-TIRADS). The Z-scores were 0.135 and 0.157, respectively, suggesting AUC differences were likely due to random variation, consistent with this study’s exploratory nature. All LLMs had significantly lower performance compared to the sonologist (p < 0.05). Additional diagnostic metrics (sensitivity, specificity, PPV, and NPV) are in **Table 1**. **Table 1** presents diagnostic performance metrics (sensitivity, specificity, PPV, NPV, and AUC) with 95% CIs for GPT-4o, GPT-o3-mini, DeepSeek-R1, and expert readings under ACR-TIRADS and C-TIRADS. ROC curves are in **Figure 3**.

Beyond these diagnostic metrics,

### 3.6 Expert evaluation of clinical recommendations

Expert assessments using 5-point Likert scales showed variability in the clinical management recommendations across LLMs. Evaluations covered five domains: guideline adherence, patient safety, operational feasibility, clinical applicability, and overall performance. Two senior clinicians (an endocrinologist and a thyroid surgeon, each with over 10 years of experience) independently evaluated the recommendations.

DeepSeek-R1 with C-TIRADS received the highest ratings across most domains.

**Table 3**, **Figure 4** present expert evaluations, showing mean Likert scores for guideline adherence, patient safety, operational feasibility, clinical applicability, and overall performance across ACR-TIRADS and C-TIRADS for DeepSeek-R1, GPT-4o, and GPT-o3-mini, as assessed by surgeons and endocrinologists. Among surgeons, DeepSeek-R1 with C-TIRADS had the highest average score (4.66), slightly outperforming DeepSeek-R1 with ACR-TIRADS (4.63). Among endocrinologists, DeepSeek-R1 with ACR-TIRADS scored slightly higher (4.29) than its C-TIRADS counterpart (4.26), indicating consistent performance across frameworks. These results suggest DeepSeek-R1 offers conservative, guideline-compliant, and clinically feasible recommendations, especially in surgical contexts. Although GPT-4o had the highest diagnostic accuracy for malignancy, its management recommendations were rated slightly lower than DeepSeek-R1’s, especially for patient safety and clinical implementation under C-TIRADS. This highlights a key insight: superior diagnostic performance does not ensure optimal clinical decision-making. GPT-4o excels in malignancy prediction, but DeepSeek-R1 is more aligned with practical clinical needs, including interpretability, safety, and guideline adherence.

**Table 3:** Five-dimensional mean scores (surgeon vs. endocrinologist).

Guideline	Model	Role	Guideline_ adherence	Patient_ safety	Operational_ feasibility	Clinical_ applicability	Overall_ performance
ACR-TIRADS	DeepSeek-R1	Surgeon	4.7460	4.6508	4.6032	4.6032	4.5714
ACR-TIRADS	GPT-4o	Surgeon	4.5556	4.5873	4.5397	4.5238	4.5556
ACR-TIRADS	GPT-o3-mini	Surgeon	4.4921	4.4603	4.4286	4.4127	4.4603
C-TIRADS	DeepSeek-R1	Surgeon	4.7778	4.6349	4.6190	4.6349	4.6508
C-TIRADS	GPT-4o	Surgeon	4.6190	4.5556	4.5238	4.5556	4.5556
C-TIRADS	GPT-o3-mini	Surgeon	4.6190	4.5397	4.5238	4.5397	4.5714
ACR-TIRADS	DeepSeek-R1	Endocrinologist	4.5397	4.3016	4.3016	4.1746	4.1270
ACR-TIRADS	GPT-4o	Endocrinologist	4.5397	4.0635	4.1111	4.0000	4.0159
ACR-TIRADS	GPT-o3-mini	Endocrinologist	4.6032	4.0000	3.9841	3.9524	3.9365
C-TIRADS	DeepSeek-R1	Endocrinologist	4.5873	4.1905	4.2381	4.1429	4.1270
C-TIRADS	GPT-4o	Endocrinologist	4.6508	4.1111	4.1270	4.0952	4.0952
C-TIRADS	GPT-o3-mini	Endocrinologist	4.6508	4.0476	4.0476	3.9524	4.0159

Scores are presented as mean values based on a 5-point Likert scale (1 = strongly disagree, 5 = strongly agree). Evaluation dimensions include Guideline Adherence, Patient Safety, Operational Feasibility, Clinical Applicability, and Overall Performance. Assessments were conducted by two types of medical professionals—surgeons and endocrinologists—on three AI models (DeepSeek-R1, GPT-4o, and GPT-o3-mini) under two thyroid nodule risk stratification systems: ACR-TIRADS and C-TIRADS.

**Figure 4:**
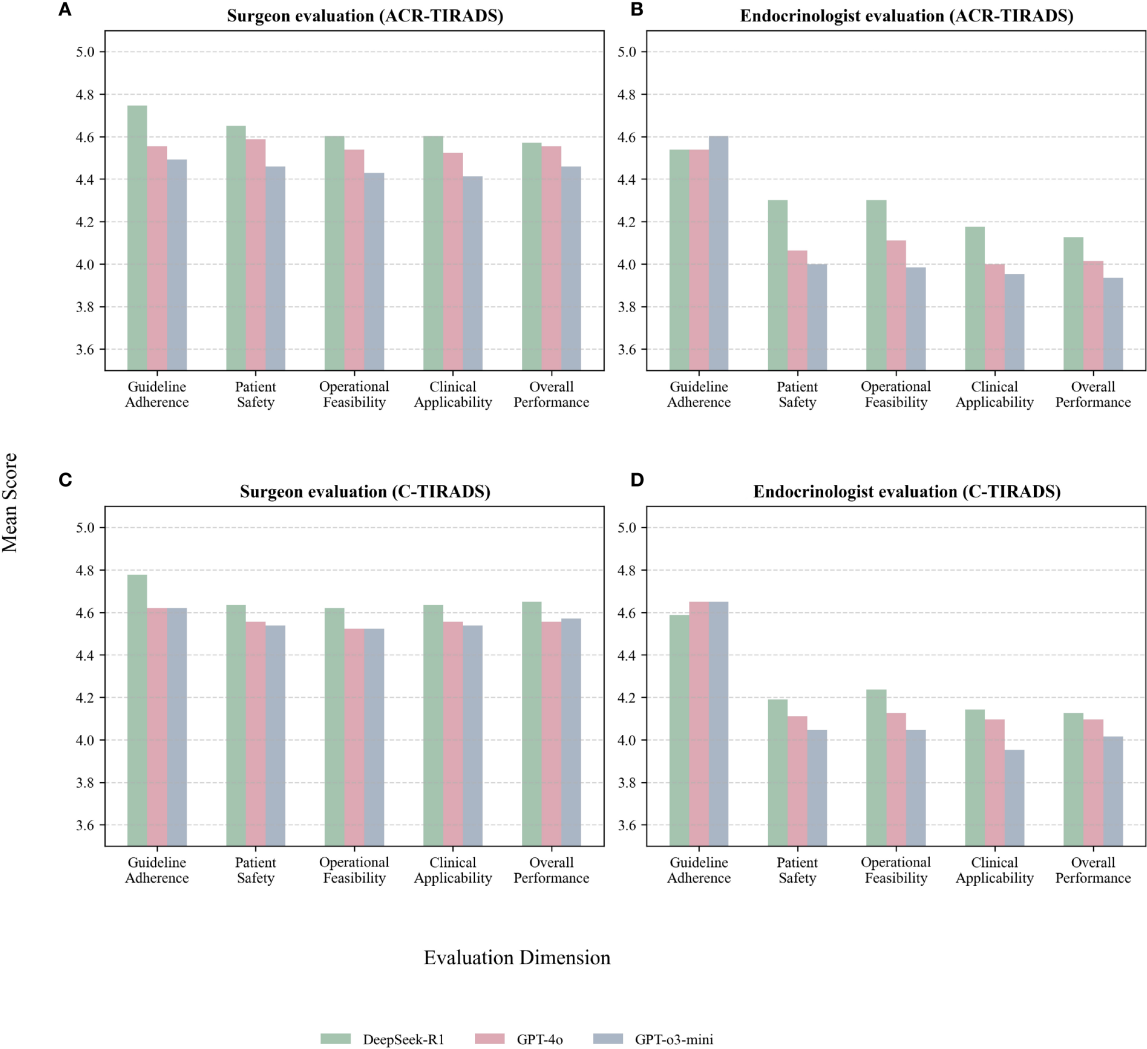
**(A)** Surgeon evaluations under ACR-TIRADS; **(B)** Endocrinologist evaluations under ACR-TIRADS; **(C)** Surgeon evaluations under C-TIRADS; **(D)** Endocrinologist evaluations under C-TIRADS.

Both experts, especially the surgeon, gave higher ratings to all models under C-TIRADS. This may reflect C-TIRADS’s simpler structure and conservative thresholds, enhancing compatibility with LLM applications.

### 3.7 Subgroup analysis

We performed subgroup analyses to evaluate model performance across benign vs. malignant nodules, nodule sizes (<10mm, 10≦20mm, ≧20mm), multifocal vs. solitary nodules, and cases with vs. without lymph node metastasis. Metrics (AUC, sensitivity, specificity, PPV, NPV, and 95% CIs) are in **Supplementary Table S2**. Performance was consistent, with no significant AUC differences between models (DeLong test, p > 0.05 in most comparisons). Sensitivity was higher in malignant and lymph node-positive subgroups, while specificity varied more in smaller and benign nodules. It should be noted that some subgroup analyses, particularly for lymph node metastasis, were limited by small sample sizes, and their results should be interpreted with caution.

### 3.8 Inter-rater reliability of expert evaluations

We assessed consistency between surgeon and endocrinologist ratings using weighted Cohen’s kappa (quadratic weights) on merged Likert scores (1–2: Low, 3: Medium, 4–5: High). This was done across all dimensions, separately for ACR-TIRADS and C-TIRADS, and per model (**Supplementary Table S3**). Inter-rater reliability showed fair consistency (C-TIRADS: mean weighted kappa = 0.277; ACR-TIRADS: 0.267) across dimensions.GPT-o3-mini under C-TIRADS had the highest agreement (mean kappa=0.380, fair), while DeepSeek-R1 showed poor agreement (C-TIRADS: -0.017; ACR-TIRADS: -0.022) due to near-uniform high ratings (~95% High), limiting score variability and kappa sensitivity. These findings indicate moderate agreement between raters, with DeepSeek-R1’s high ratings reflecting strong perceived reliability but reduced discriminatory power in consistency metrics.

### 3.9 Error analysis of model performance

Error analysis showed that models struggled with nodules having subtle or complex features, such as mixed calcifications, small size, or ambiguous boundaries. These features caused inconsistent malignancy risk predictions, often leading to over- or underestimation. These findings highlight the need for clinician oversight in ambiguous cases, as LLMs may not reliably distinguish nuanced features without expert input. This underscores the importance of human-AI collaboration in high-stakes decisions. Error analysis showed model failures in small nodules (<10mm), with AUC ~0.53-0.55 and lower specificity, risking over-diagnosis. In multifocal or lymph node-positive cases, sensitivity declined (e.g., GPT-4o: 0.870), potentially missing malignancies. These patterns suggest increased human oversight for ambiguous features. Future work should address rare subtypes through multimodal improvements.

## 4 Discussion

### 4.1 Principal findings

We evaluated the clinical recommendation capabilities of three LLMs (GPT-4o, GPT-o3-mini, and DeepSeek-R1) using ACR-TIRADS and C-TIRADS frameworks.

GPT-4o achieved the highest diagnostic accuracy for malignancy prediction via ROC analysis. However, DeepSeek-R1 with C-TIRADS received the highest ratings from endocrine and surgical experts. These assessments, across five domains (guideline adherence, patient safety, operational feasibility, clinical applicability, and overall performance), highlight both content accuracy and clinical practicality of model recommendations. This aligns with Topcuoglu (15) and Jin et al. (16), who highlight C-TIRADS’s greater specificity and biopsy utility for Chinese populations compared to ACR-TIRADS. DeepSeek-R1’s higher expert ratings may reflect its alignment with regional clinical reasoning and linguistic context, as noted by Chen (7) and Gibney (17). The discrepancy between GPT-4o’s higher AUC (0.898) under C-TIRADS and DeepSeek-R1’s higher clinician ratings (surgeon: 4.65; endocrinologist: 4.63) suggests a divergence between objective accuracy and subjective clinical utility. AUC measures overall balance, with GPT-4o’s high specificity (0.927) reducing false positives. In contrast, DeepSeek-R1’s high sensitivity (0.957) prioritizes malignancy detection, fostering greater trust in patient safety, a key rating dimension. DeepSeek-R1’s regional origins may enhance C-TIRADS alignment, improving perceived adherence and operational fit. These suggest tailored LLM use: GPT-4o for accuracy-driven screening and DeepSeek-R1 for safety-critical or region-specific decision support.

A key insight is the disconnect between diagnostic performance and clinical trustworthiness. This gap underscores the importance of interpretability and usability for LLM deployment. Fair inter-rater reliability between the surgeon and endocrinologist (C-TIRADS: mean weighted kappa=0.277; ACR-TIRADS:0.267; **Supplementary Table S3**) suggests potential bias from using only two raters. DeepSeek-R1’s near-zero kappa (C-TIRADS: -0.017; ACR-TIRADS: -0.022) results from near-uniform high ratings (~95% High). This indicates strong clinician agreement but limited score variability, reducing kappa sensitivity. This underscores the need for diverse raters (e.g., radiologists, additional specialists) in future studies to improve reliability and generalizability, consistent with multi-center validation needs (18).

We observed variability in model performance across subgroups, particularly in AUC, sensitivity, and specificity. Smaller subgroup sample sizes (e.g., lymph node metastasis: Yes, Pathology = 0) caused performance differences, highlighting limitations of small datasets. These findings highlight the need for future research with larger, more diverse cohorts to enhance model robustness across subgroups.

Subgroup analyses (**Supplementary Table S2**) showed diagnostic challenges in smaller nodules (<10mm) and benign cases. Lower AUC (e.g., expert: 0.546 under ACR-TIRADS) and variable specificity suggest increased false positives, likely due to subtle ultrasound features (19). This aligns with studies showing that small nodules pose management challenges, with lower TIRADS diagnostic accuracy, requiring multimodal approaches for better differentiation (19). Multifocal nodules had slightly higher AUC than solitary nodules, likely due to richer feature sets for risk stratification. This is consistent with evidence linking multifocal disease to higher malignancy rates and complexity (20). In lymph node metastasis cases, high sensitivity but lower specificity was observed, suggesting potential over-diagnosis in aggressive cases. This aligns with ultrasound studies noting imaging’s role in detecting, but sometimes overcalling, metastatic features (20). These findings highlight the need for refined AI models with multimodal data to address subgroup-specific challenges, especially in settings where small, benign, or non-metastatic nodules predominate. DeepSeek-R1 with C-TIRADS received the highest clinician ratings, particularly for guideline adherence, patient safety, and operational feasibility. Its alignment with regional guidelines and structured decision-making resonates with clinicians, especially surgeons. Despite GPT-4o’s higher AUC under C-TIRADS, clinicians rated it lower, likely due to less intuitive outputs compared to DeepSeek-R1’s actionable, guideline-aligned recommendations. Clinician trust often outweighs AUC in AI model adoption, as usability and alignment with clinical reasoning are critical for clinical implementation. Doshi et al. (21) showed that context-specific prompting, like simplifying radiology reports for laypersons, improves comprehension without compromising accuracy.

These results align with existing literature.

### 4.2 Comparison with previous studies

Although research has explored LLMs in medical reasoning and imaging interpretation, few studies have examined their alignment with region-specific guidelines or physician expectations.

LLM performance in thyroid imaging varies. Wu et al. (22) found GPT-4.0 improved diagnostic consistency and sometimes outperformed junior clinicians. In contrast, Chen et al. (23) reported low concordance with pathological findings and lower accuracy than.

radiology trainees. Katharina et al. (24) noted GPT-4.0’s difficulty adhering to ACR-TIRADS, highlighting limitations in handling complex protocols.

Model performance varies significantly. Kaba et al. (25) reported GPT-4’s strong performance with K-TIRADS, while Xia (26) and Wang (11) found GPT-3.5 useful for general queries but unreliable for nuanced decisions. Marchi et al. (27) and Chung et al. (28) concluded that, while LLMs aid treatment planning and risk stratification, domain-specific models outperform in high-stakes settings.

DeepSeek-R1 shows promise in recent studies. Peng (29) and Liang (30) showed DeepSeek-R1’s effective adaptation to clinical reasoning in Chinese healthcare settings, consistent with its strong performance under C-TIRADS. These results suggest that language proficiency or model size alone is insufficient; alignment with local practice patterns and clinical logic is critical. Chen et al. (30) proposed a multi-agent, GPT-4–based framework that improved diagnostic accuracy and follow-up planning for rare diseases. This LLM collaboration model highlights the potential for multi-agent systems in complex diagnostic.

Our findings align with advancements in AI for thyroid nodule diagnosis, notably the ThyGPT model, which integrates ultrasound imaging with LLMs to aid radiologists in risk stratification and decision-making. Yao et al. (7) showed that ThyGPT significantly improves diagnostic accuracy, surpassing traditional methods in sensitivity and specificity. ThyGPT’s ability to detect and correct ultrasound report errors underscores its potential as a reliable AI copilot, aiding radiologists and reducing diagnostic errors and unnecessary procedures. Grani et al. (3) support AI’s growing role in enhancing diagnostic accuracy via AI-driven models, particularly compared to traditional systems like ACR-TIRADS and C-TIRADS.

#### 4.2.1 Comparison of TIRADS systems

Alongside LLMs’ varied performance in thyroid imaging, multiple ultrasound risk stratification systems (e.g., ACR-TIRADS, EU-TIRADS, K-TIRADS) are widely used in clinical settings. These systems, differing in risk classification and regional applicability, show varied diagnostic performance. Kim et al. (18) and Piticchio et al. (31) showed performance differences across these systems, particularly in different regions. K-TIRADS, widely used in Asia, often classifies more nodules as high-risk than ACR-TIRADS and EU-TIRADS, which are common in Western countries and show more moderate classifications. These regional differences are key to understanding TIRADS application in clinical practice and tailoring diagnostic strategies to specific populations.

### 4.3 Implications for clinical practice and AI deployment

Our findings have key implications for the integration of LLMs into clinical workflows:

• Localization and context-aware prompting are essential. Yang et al. (32) noted that adapting to regional guidelines is as critical as model architecture or scale. Our results confirm this, showing DeepSeek-R1 outperformed larger models in C-TIRADS due to better contextual alignment. Bayesian-adjusted PPV and NPV values at different prevalence levels (5%, 10%, 15%) highlight the need to consider clinical prevalence in evaluating AI model applicability.

Cognitive congruence fosters clinical trust. Higher expert ratings for C-TIRADS outputs suggest clinicians prefer LLM recommendations mirroring their reasoning and decision-making frameworks. Kaba et al. (25) noted that interpretability and familiarity are key for AI systems trust.

- Role-based customization reduces trust gaps. Clinician attitudes toward DeepSeek-R1 and GPT-4o under C-TIRADS vary by role. The surgeon preferred DeepSeek-R1 (e.g., overall performance 4.65 vs. 4.56 for GPT-4o), likely valuing its high sensitivity (0.957) for confident surgical decision-making and risk mitigation. The endocrinologist rated both lower (DeepSeek-R1: 4.13; GPT-4o: 4.10), likely prioritizing specificity for monitoring and avoiding unnecessary procedures, consistent with endocrine practice. Poor inter-rater agreement (weighted kappa: -0.017 for DeepSeek-R1; **Supplementary Table S3**) reflects these differences. The ‘kappa paradox’—near-uniform high ratings (~95% High)—limits variability, resulting in low kappa despite strong observed agreement. This suggests customizing LLMs: sensitivity-focused for surgeons and specificity-focused for endocrinologists to reduce the trust gap.
- Fine-tuning and clinician-centered design promote adoption (33): Strategies like reinforcement learning with human feedback (RLHF), localized instruction tuning, and guideline-informed prompt engineering improve LLM output utility. Our findings support clinician-aligned AI development to ensure safe and effective deployment.

These insights advocate for future LLM systems to be designed around three principles: contextual alignment, transparency, and collaborative augmentation of clinician expertise 95% CI.

### 4.4 Strengths and limitations

A key strength is the use of real-world, diagnostically challenging ultrasound cases rather than synthetic scenarios, enhancing ecological validity. The inclusion of endocrinologists and surgeons in evaluations provides a multidisciplinary perspective, adding depth and practical relevance. Several limitations should be noted.

A limited number of expert raters: A key limitation is relying on only two evaluators (a surgeon and an endocrinologist), which may introduce bias and limit generalizability of Likert-scale ratings. Inter-rater reliability, assessed via weighted Cohen’s Kappa (**Supplementary_Table_S3**), shows fair to moderate agreement (e.g., 0.261 for C-TIRADS). Low or negative values reflect the ‘kappa paradox’ due to high rating uniformity. Future prospective studies with larger, diverse multidisciplinary panels (e.g., ≧6raters) could improve validity, as recommended in LLM healthcare evaluations (7, 34).

- Selection bias: We intentionally curated cases for diagnostic ambiguity. While useful for stress-testing models, this may not reflect routine clinical presentations. The subset (n=63) aligns demographically with the total cohort (**Table 2**; p > 0.05 for gender, age, nodule size) and general populations (median age: 52 years; 71% female) but has higher malignancy (73% vs. 45.5%, p < 0.001), limiting generalizability to low-risk screenings (35). Bayesian-adjusted PPV/NPV for 5–15% prevalence (**Supplementary Table S1**) supports applicability in routine settings. High NPV (>0.96) aids benign nodule exclusion, reducing biopsies, while lower PPV (0.22–0.65) may increase false positives, consistent with malignancy rates of 7–15% (36). Larger, balanced cohorts are required. DeLong tests (p>0.05; **Supplementary Table S2**) confirm no significant AUC differences, supporting result reliability despite the modest sample size. We plan prospective studies with larger cohorts (e.g., n > 200) to validate findings with predefined power calculations, improving applicability to diverse populations.
- Single-center design: All clinicians were from one Chinese institution, potentially introducing institutional and regional bias. Clinical judgment and guideline interpretation vary across healthcare systems due to differences in training, resources, and cultural norms. Multicenter studies across diverse hospital tiers and regions are needed to improve external validity. Future multicenter studies with diverse raters will improve generalizability.
- Non–real-time testing: We evaluated LLMs offline, not in live clinical environments. Thus, the real-world impact of LLM recommendations on workflow efficiency, patient safety, and decision-making is uncertain. Prospective validation in operational settings is required. Future real-time testing will evaluate practical utility in clinical workflows.
- High-consensus data annotation: Our three-expert agreement process ensured consistency and data integrity but is resource-intensive, potentially limiting scalability in routine practice.

Lack of multimodal input: We prompted models with textual ultrasound descriptions rather than raw images. Although this reflects current documentation practices, it excludes rich visual information used by sonographers. Multimodal models like ThyGPT (7) could improve accuracy by integrating visuals, reducing biopsies by >40% in similar studies. Future multimodal models integrating text and images will improve diagnostic capability, pending ethical approvals. Future work should explore multimodal or vision-language models integrating narrative and image data to improve diagnostics, especially in complex or ambiguous cases. Subgroup analysis (**Supplementary Table S2**) shows model vulnerabilities in small nodules (<10mm), with AUC ~0.53–0.55 and lower specificity, increasing over-diagnosis risk. In multifocal or lymph node-positive cases, sensitivity declined (e.g., GPT-4o: 0.870), potentially missing malignancies. These patterns suggest increased human oversight for ambiguous or rare features, highlighting AI as an adjunct tool. Future multimodal integrations could address these limitations. • Addressing these limitations will strengthen future research.

## 5 Summary

In clinical settings, usability and clinician trust in model recommendations often outweigh AUC for AI adoption. This underscores the need to consider clinical feasibility and trust alongside diagnostic performance for AI implementation.

## Data Availability

The original contributions presented in the study are included in the article/**Supplementary Material**. Further inquiries can be directed to the corresponding author.

## Supplementary material

Supplementary Table 1 Bayesian-adjusted positive predictive value (PPV) and negative predictive value (NPV) for malignancy prediction at varying prevalence rates (5%, 10%, 15%) across models and TIRADS frameworks.

Supplementary Table 2 Subgroup performance metrics and DeLong AUC comparisons for models under ACR-TIRADS and C-TIRADS.

Supplementary Table 3 Weighted Cohen’s Kappa values for inter-rater reliability between surgeon and endocrinologist evaluations across dimensions (guideline adherence, patient safety, operational feasibility, clinical applicability, overall performance), models, and TIRADS frameworks.
